# NK Cell and CD4+FoxP3+ Regulatory T Cell Based Therapies for Hematopoietic Stem Cell Engraftment

**DOI:** 10.1155/2016/9025835

**Published:** 2016-01-05

**Authors:** Antonio Pierini, Maite Alvarez, Robert S. Negrin

**Affiliations:** ^1^Blood and Marrow Transplantation, Stanford University School of Medicine, Stanford, CA 94305, USA; ^2^Hematology and Clinical Immunology, Department of Medicine, University of Perugia, 06132 Perugia, Italy

## Abstract

Allogeneic hematopoietic cell transplantation (HCT) is a powerful therapy to treat multiple hematological diseases. The intensive conditioning regimens used to allow for donor hematopoietic stem cell (HSC) engraftment are often associated with severe toxicity, delayed immune reconstitution, life-threatening infections, and thus higher relapse rates. Additionally, due to the high incidence of graft versus host disease (GvHD), HCT protocols have evolved to prevent such disease that has a detrimental impact on antitumor and antiviral responses. Here, we analyzed the role of host T and natural killer (NK) cells in the rejection of donor HSC engraftment as well as the impact of donor regulatory T cells (Treg) and NK cells on HSC engraftment. We review some of the current strategies that utilize NK or Treg to improve allogeneic HCT therapy in order to accomplish better HSC engraftment and immune reconstitution and achieve a lower incidence of cancer relapse, opportunistic infections, and GvHD.

## 1. Introduction

Hematopoietic cell transplantation (HCT) is the only curative treatment for high-risk leukemias and lymphomas and for several nonmalignant hematologic diseases such as hemoglobinopathies and severe combined immune deficiencies. Unfortunately, one of the major barriers to this therapeutic approach is the rejection of the stem cell graft by the host immune system. The highest donor engraftment rate is obtained in autologous HCT when the patient's hematopoietic stem cells (HSCs) are previously collected and successively reinfused after the conditioning regimen. However, the incidence of cancer relapse after autologous HCT for some diseases such as acute myeloid leukemia is high and thus allogeneic HCT is a more desired option [1, 2]. Differences in human leukocyte antigen (HLA) haplotypes between donor and host may trigger rejection through host versus graft immune reactions [3]. Myeloablative conditioning regimens that can include total body irradiation or high dose chemotherapy eliminate most of the host immune system allowing for donor stem cell engraftment in several preclinical models and clinical studies [4–8]. As HLA-matched donors were often unavailable, during the 1980s and 1990s, several investigators explored ways to promote engraftment in HLA-mismatched conditions where host versus graft reactions are stronger (e.g., following haploidentical HCT) and where fully myeloablative conditioning was often not enough to avoid rejection [9, 10]. The infusion of a “megadose” of stem cells from the donor and their “veto” effect coupled with depletion of residual radio- and chemoresistant host T cells* in vivo* allowed for successful HSC engraftment even in these challenging clinical situations [11–14]. Despite these clinical advancements and strategies that made HCT available to most of the patients that required it, they have still resulted in a number of significant complications [15]. Elderly patients and patients with comorbidities often cannot tolerate myeloablative conditioning regimens and* in vitro* and/or* in vivo* T cell depletion is responsible for poor immune reconstitution after HCT often leading to severe and life-threatening infections. Moreover, children with nonmalignant diseases that require HCT need to be treated with the minimal toxic conditioning regimen that allows for donor stem cell engraftment. It is clear that new therapeutic approaches are needed to perform HCT in these subsets of patients.

Recent studies have highlighted the role of the different immune cells in rejection. The discovery of cells with regulatory and tolerogenic properties opened the possibility of new treatments for inducing tolerance to HSC engraftment and reducing the use of toxic therapies. In this review, we will focus on the role of natural killer (NK) cells and CD4+FoxP3+ regulatory T cells (Treg) in donor HCT engraftment and immune tolerance. New studies on NK cells highlighted the existence of different subsets that possess different characteristics and can be modulated to promote engraftment. Furthermore, Treg have been widely studied for their tolerogenic properties and their ability to suppress conventional T cells (Tcon) and other immune cells such as NK and B cells* in vitro* and* in vivo*; therefore, they hold tremendous promise for clinical application in the HCT setting.

## 2. Stem Cell Rejection: Role of Host NK and T Cells

In the first experiences of bone marrow transplantation rejection appeared as an insurmountable barrier. Many attempts have been made to overcome this issue and promote donor HSC engraftment which have been illustrative to uncover the cells and mechanisms involved in graft rejection. Residual host Tcon play a key role in this process; they recognize HLA antigens of the donor cells and organize an immune attack against donor HSC leading to rejection [3, 16–18]. Several studies demonstrated that T cells are chemo- and radioresistant cell population; therefore, their* in vivo* depletion through the use of selective drugs against T cells such as antithymocyte globulin (ATG) was required, even if not always enough, to induce engraftment in HLA-mismatched patients [19]. T cell mediated immune reactions are potent when donor and host are mismatched on HLA antigens. While these conditions lead easily to HSC rejection, donor T cell infusion can overcome the problem inducing engraftment through strong graft versus host reactions, but at the same time increasing the risk of graft versus host disease (GvHD), a potentially lethal complication caused by a donor attack to the host tissues. Donor T cells can recognize HLA antigens on host cells and tissues resulting in immune attack and leading to life-threatening GvHD [20]. T cell depletion of the donor graft dramatically reduces GvHD incidence, but it may also limit donor HSC engraftment [8, 21, 22].

CD4+ and CD8+ subsets of T cells play a role in the induction of T cell mediated rejection, but the few residual CD8+ T cells that survive the conditioning regimen seem to be the main responsible population [23–25]. A rapid increase of T cells coupled with a likewise rapid loss of donor chimerism in peripheral blood of transplanted patients is often seen during rejection [26, 27]. Host T cells are triggered by non-self-HLA antigens, are activated, and respond against donor HSCs, but the mechanisms through which residual host Treg fail to prevent this immune reaction are not fully understood. It is relevant in this context that Treg not only can regulate T cells, but are able to suppress function of several immune cells, including NK cells; therefore, Treg-based treatments are under investigation in an effort to control T cell and NK cell mediated HSC rejection.

Historically, NK cells have been shown to play a key role in limiting HSC engraftment ability after HCT. Indeed, one of the first observations of NK cell activity in this context showed that NK cells were able to eliminate parental and allogeneic allografts from lethally irradiated hybrid F1 mice leading to the concept of “hybrid resistance” [28]. Ljunggren and Karre in the 1980s established that NK cells could eliminate cells that fail to present self-MHC class I molecules proposing the “missing self” hypothesis and therefore being the first to provide a mechanism for the role of NK cells in the rejection of allogeneic HSC [29]. The later identification of inhibitory receptors on NK cells, which contain immunoreceptor tyrosine-based inhibitory motifs (ITIM) in the cytoplasmic domain, revealed the system by which the recognition of MHC class I molecules results in NK cell inhibition [30, 31]. In mice, most of the inhibitory receptors fall into the Ly49 family, whereas in humans they are called killer immunoglobulin-like receptors (KIRs). Though structurally different, both types of inhibitory receptors share the same function, which is to regulate NK cell activation [32]. Due to the stochastic expression of inhibitory receptors on NK cells [32], the NK cell repertoire is composed of NK cells that express inhibitory receptors that bind to both self- and/or non-self-MHC class I molecules. Kim et al. proposed the NK cell “licensing” hypothesis and showed evidence of how NK cells express inhibitory receptors for self-MHC class I molecules: licensed NK cells are more functionally competent that those NK cells that express inhibitory receptors for non-self-MHC class I or unlicensed NK cells [33]. Indeed, NK cells developing in MHC class I deficient mice show lower NK cell function than their counterparts developed in MHC class I competent recipients [33]. The concept of “licensing,” “arming,” or “education” was confirmed by other groups for both human and mouse NK cells [34, 35].

**Figure 1:**
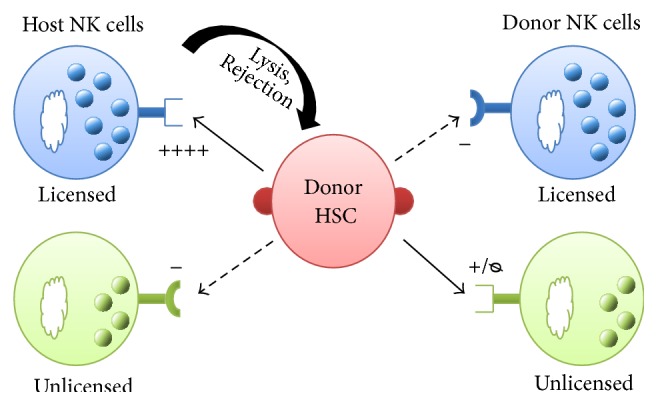
Role of NK cells during allogeneic HSC rejection. Donor allogeneic HSCs express MHC class I molecules that are not recognized by the inhibitory receptors of licensed NK cells resulting in HSC lysis, whereas there can be unlicensed NK cell subsets that express inhibitory receptors capable of recognizing the MHCI of allogeneic BMC. On the contrary, donor NK cells share MHCI expression with donor BMC and thus are less likely to play a role in allogeneic BMC rejection. In allogeneic HCT settings, the presence of host licensed NK cells at the time of transplantation can influence the donor engraftment outcome.

Taking into consideration the importance of NK cell licensing in the acquisition of NK cell function, it makes sense to hypothesize that this mechanism also plays a role in the rejection of HSC after allogeneic HCT. It has been shown that NK cells are also a radioresistant population [36, 37]. Thus, the timing of bone marrow infusion after lethal radiation is critical to understand the role of host NK cells in HSC engraftment. In fact, depletion of host NK cells prior to HSC infusion results in enhanced allogeneic HSC engraftment [38]. In order to segregate the role of NK cell subsets, experiments where different NK cell subsets were depleted prior to allogeneic HCT showed that host licensed NK cells were indeed the main cell population responsible for allogeneic BMC rejection limiting engraftment, whereas host unlicensed NK cell subsets had little or no role [38]. Taking into consideration that donor NK cells were educated in the presence of donor MHC, no rejection of donor engraftment is expected from unlicensed donor NK cells and therefore host licensed NK cells play the major role in allogeneic HCT rejection (Figure 1). Thus, the selection of a particular NK cell subset as well as the timing of HCT can be useful to control donor HSC engraftment outcome. Moreover, the degree of NK cell activation prior to allogeneic HCT is an important parameter to consider. Sun et al. elegantly demonstrated that licensed NK cell subsets were able to produce higher amounts of cytokines than unlicensed NK cells, but they also showed that upon activation the hyporesponsiveness of unlicensed NK cells is resolved and this subset is perfectly functional. Additionally, if host NK cells were stimulated using cytokines or poly I:C before HCT, only depletion of the whole NK cell population or depletion of both licensed and unlicensed NK cell subsets was able to facilitate allogeneic HSC engraftment [38].

Another important parameter that can affect the role of NK cell subsets in the rejection of allogeneic BMC is the binding affinity of the inhibitory receptors for multiple MHC class I molecules. Hanke et al. showed that, except for the mouse Ly49G2 inhibitory receptors, Ly49 inhibitory receptors can have different levels of binding affinity for their ligands [39], resulting in some degree of licensing. An example of this versatility can be found in the Ly49A inhibitory receptor which has the highest affinity for the H2^d^ haplotype, making the NK cells that carry it licensed in H2^d^ strains, but which can also bind other MHC class I molecules with lower affinity [39]. Andrews et al. demonstrated that Ly49A can bind to H2-M3, a nonclassical MHC class I molecule constitutively expressed on B cells, and regulate its licensing pattern. They showed that in H2^b^ mice Ly49A^+^ NK cells display some degree of licensing despite being previously thought to be unlicensed [40]. Similar versatility can also be found in human KIRs. For example, KIR2DL2, unlike other KIRs, is capable of binding many HLA-C alleles products with variable affinities [41, 42]. It is possible that polymorphic residues that alter the affinities between KIR and its HLA ligands impact the degree of NK cell licensing and thus their effector functions similar to Ly49A in mouse [43].

In addition to the degree of licensing, the presence of immunosuppression at the time of HCT can also impact the allogeneic HSC engraftment ability in the presence of NK cells. It is known that the immunosuppressive cytokine transforming growth factor-beta (TGF-*β*) can inhibit NK cells by reducing IFN-*γ* production, degranulation, and overall cytotoxic functions [44–46]. In fact, exogenous administration of TGF-*β* was shown to modify the inhibitory and activating receptor balance by lowering the levels of NKG2D and NKp30 activating receptors and thus hampering IFN-*γ* production [46]. Depletion of host Treg, an important source of TGF-*β*, induced stronger NK cell-dependent allograft rejection, demonstrating the role of TGF-*β* in the suppression of NK cell activity [47].

In summary, both host T and NK cells have a crucial role in inducing HSC rejection. Modulation of environmental stimulation, subset ratios, and immunosuppressive agents may result in relevant modifications to host T and NK cell mediated immune responses, thus impacting HCT outcome.

## 3. Donor NK Cells for Promotion of Engraftment

As mentioned above, donor T cell infusion increases GvT and allogeneic HSC engraftment but also induces GvHD. Unfortunately, many treatments that reduced GvHD severity were often accompanied by decreased GvT effects and cancer relapse [48]; therefore, alternative strategies that did not involve donor T cell infusion have been studied.

NK cells, as we have already stated, play a critical role in donor HSC engraftment due to NK cell licensing. They are also known to be the first lymphocyte population that reconstitutes after HCT. Some studies have suggested that NK cells display a more immature phenotype with impaired function shortly after transplantation [49]. However, others suggest that NK cells become active due to the inflammatory cytokine milieu that follows the conditioning regimen. In addition, NK cells have the important advantage that their alloreactivity is restricted to HSCs and there is not an association between NK cells and GvHD induction [50]. Therefore, selection of donor NK cell subsets, similar to the selection of host NK cell subsets, can also impact the outcome of engraftment and antitumor responses without affecting GvHD. In particular, in the haploidentical setting, NK alloreactive donors can induce extremely strong antitumor responses highly reducing relapse rate in patients with acute myeloid leukemias. Yu et al. [51] showed that unlicensed NK cells, defined as those NK cells that lack inhibitory KIRs for donor MHC ligands, become competent and alloreactive when the ligand was also missing in the host in both HLA-mismatched and HLA-matched settings [51]. Yu et al. also demonstrated that, during HLA-matched HCT, these alloreactive unlicensed NK cells were initially able to break tolerance to self and gradually become tolerant to the self-acquiring of the known unlicensed hyporesponsive donor phenotype by day +200 after HCT [51]. This NK cell tolerance to self was also observed in early studies where tolerance was achieved during the first year after HCT in haploidentical transplants with KIR-ligand incompatibility and isolation of alloreactive donor NK cells was rare after the first three months following transplant [52]. Similarly, allogeneic HCT studies in both human and mouse confirmed that NK cell development also resembles the donor-type NK cell licensing pattern [51, 53, 54].

Importantly, shortly after HLA-mismatched HCT, alloreactivity can be accomplished from NK cells that express KIRs for HLA ligands other than the host HLA and thus, due to the HLA mismatch, it can be sustained by both licensed and unlicensed NK cells. Due to the already described phenomenon of expansion and activation of unlicensed NK cells shortly after transplantation in both human and mouse studies [51, 55, 56], it is likely that activated alloreactive unlicensed donor NK cells collaborate with alloreactive licensed donor NK cells in the initial clearance of host HLA-expressed tumor cells while impacting the degree of allogeneic engraftment. The presence of alloreactive licensed NK cells can also play a fundamental role in the engraftment of donor HSCs allowing for overall acceleration of HSC reconstitution. Early studies demonstrated that the infusion of activated donor-type NK cells enhanced donor cell engraftment and development during allogeneic HCT [57–59]. Indeed, the selective infusion of alloreactive NK cells following allogeneic HCT during nonmyeloablative and myeloablative conditioning allowed for durable donor-type chimerism, even in donor grafts containing T cells, resulting in a reduced incidence of GvHD [50]. It has been proposed that alloreactive NK cells control GvHD incidence by the elimination of host-type antigen presenting cells (APCs) which prevents the priming of donor alloreactive T cells and thus GvHD [50] consistent with the role of NK cells in the homeostatic regulation of APCs during viral infections [60, 61]. Furthermore, Olson et al. demonstrated that activated donor NK cells can efficiently eliminate alloreactive donor T cells in NKG2D-dependent manner due to the upregulation of NKG2D ligands on the alloreactive T cell population [62]. Upregulation of Fas has also been observed in cytokine-dependent activated T cells which allows for targeting by NK cells in a FasL-dependent manner [63]. These studies suggest that NK cells can regulate alloreactive T cells through multiple mechanisms reducing GvHD incidence and lethality after allogeneic HCT. Importantly, while NK cell alloreactivity in HLA-mismatched HCT can prevent GvHD, this alloreactivity is fundamental to achieve a significant antitumor response. Ruggeri et al. demonstrated that alloreactive NK cells significantly improved engraftment with reduced incidence of GvHD resulting in an overall survival benefit in patients with AML [50].

Alternatively, some studies have suggested that the selection of activating receptors can have a significant positive impact on antitumor responses after HCT and might contribute to alloreactive NK cell function based on the donor inhibitory receptor repertoire. The presence of KIR2DS1 on NK cells from the donor graft appears to provide protection against AML relapse and KIR3DS1 was also associated with reduced mortality [64]. Indeed, a recent study by Mancusi et al. demonstrated that the KIR-ligand mismatched donors with KIR2DS1 and KIR3DS1 expression have a survival advantage during haploidentical HCT because of a reduced infection rate and mortality [65]. However, the impact of activating receptors on the donor HSC engraftment has not been elucidated yet, but we believe that the inhibitory receptor repertoire would have a relevant role in this setting.

Antibodies against KIRs have been explored in order to enhance NK cell antitumor responses. 1-7F9, a human antibody against KIR, was shown to improve HLA-matched AML blast elimination both* in vivo* and* in vitro* [66] and, in combination with lenalidomide, increased the cytotoxic functions of NK cells in MM patients [67]. The use of anti-KIR in combination with anti-CD20 also induced a significant increase of NK cell mediated, rituximab-dependent cytotoxicity against lymphoma [68]. Interestingly, Sola et al. elegantly demonstrated in a preclinical mouse model not only that the use of anti-KIR for the human inhibitory receptor KIR2DL3 was able to enhance NK cell cytotoxic function without breaking self-tolerance or inducing autoimmunity but also that the long term treatment with anti-KIR did not alter NK cell licensing [69]. During HCT, all of the NK cell subsets become highly activated shortly after transplantation because of the conditioning regimens required; therefore, the impact of an anti-KIR antibody in such a stimulatory environment might be strong.

Although the administration of anti-KIR antibodies to improve allogeneic HCT engraftment has not been evaluated yet, the use of specific anti-KIR to neutralize those NK cells that express inhibitory receptors involved in HSC rejection could significantly increase allogeneic HSC engraftment and result in antitumor, antiviral, and anti-GvHD responses.

**Figure 2:**
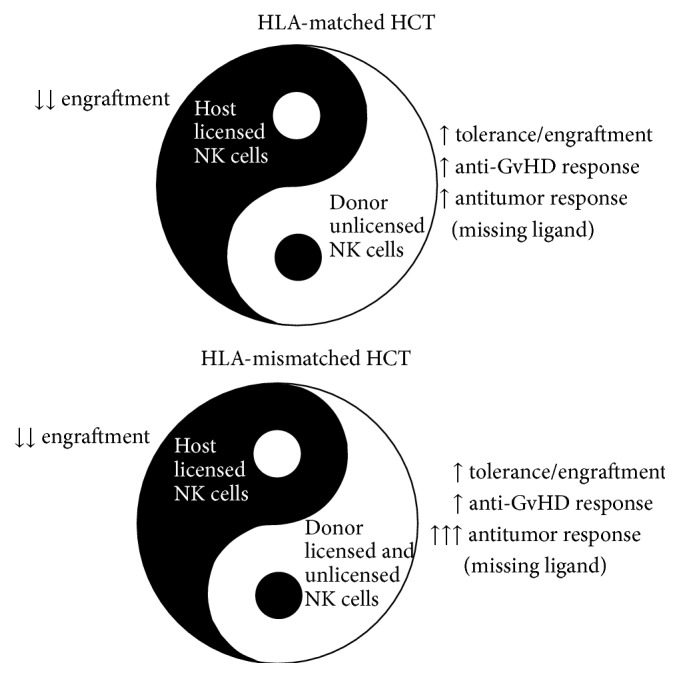
Impact of donor NK cells during allogeneic HCT. Host licensed NK cells are responsible for HSC rejection minimizing donor HSC engraftment. Donor NK cells, in contrast, are known to be tolerant to donor HSC and play a fundamental role in the reduction of GvHD by the elimination of alloreactive T cells. Donor unlicensed NK cells, in HLA-matched HCT, and licensed NK cells, in HLA-mismatched (haploidentical) HCT, can additionally provide stronger antitumor responses due to the lack of recognition of HLA in tumor cells (missing ligand).

NK cell based cellular therapies are considered a potential anticancer treatment in combination with allogeneic HCT due to NK cell mediated antitumor responses. Moreover, NK cells are capable of influencing HSC engraftment and thus play a critical role in the early immune reconstitution of patients undergoing allogeneic HCT (Figure 2). Therefore, controlling parameters such as host conditioning, HCT timing, NK cell subsets, and activation status can be optimized with the goal of maximizing allogeneic HSC engraftment and overall immune reconstitution.

## 4. Regulatory T Cells and Tolerance Induction

Since their discovery, Treg have been studied with a goal of utilizing their properties for tolerance induction in different conditions. In preclinical models of HCT, Treg have demonstrated ability to prevent GvHD while preserving graft versus tumor effects [70–74]. These results have been translated to the clinic by different groups in phase I/II clinical trials demonstrating Treg safety and potential efficacy in prevention or treatment of acute GvHD (aGvHD) and chronic GvHD (cGvHD) [75–79]. In these studies, Treg from different donor sources have been adoptively transferred to suppress donor Tcon proliferation and activity. Treg have been employed in different HCT settings and they demonstrated ability to suppress GvHD even in haploidentical HCT where donor versus host reactions are extremely potent and where few Tcon can trigger lethal GvHD. A brief summary of different clinical approaches with the use of Treg adoptive transfer in HCT setting is reported in Table 1. Other groups have also explored the possibility of enhancing Treg function* in vivo*. Treg are extremely sensitive to interleukin-2 (IL-2) stimulation and treatment with low dose IL-2 has resulted in Treg activation, expansion, and enhanced function in animal models [80–83]. The group from Dana-Farber Cancer Institute (Boston, USA) conducted a clinical trial of low dose IL-2 in patients with cGvHD. In this study, IL-2 induced expansion of Treg and NK cells, was safe, and promoted clinical improvement in 12 of 23 treated patients with steroid refractory cGvHD [84, 85]. Even if preclinical models did not clarify whether Treg-based therapies are equally effective against aGvHD and cGvHD yet, these pilot clinical studies demonstrated that therapies aiming to transfer Treg or enhance their* in vivo* function may be used for preventing or treating both GvHD clinical forms. Time of Treg injection and administered dose seem to be crucial for clinical efficacy, but differences in HCT donor source and protocols do not help to identify the best approach to be used; therefore, further studies are required to solve these critical issues.

Treg have been demonstrated to be effective in promoting engraftment of donor HSC across different MHC barriers. Joffre et al. showed that* in vitro* stimulated Treg with donor-derived APCs are able to promote engraftment after bone marrow transplantation in allogeneic conditions [86]. Pilat et al. showed that host-type Treg can induce donor chimerism after transplantation of BM cells in allogeneic conditions even in the absence of a cytotoxic conditioning regimen, by treating animals with short-course costimulation blockade (CTLA4Ig, anti-CD40L) and rapamycin [87, 88]. The ability of Treg to induce tolerance to the BM graft seems to be not dependent on MHC identity between Treg and the host environment as “third-party” Treg have been shown to limit rejection mediated by host-type Tcon [89]. Furthermore, Treg induce HSC cell cycling and contribute to building a hematopoietic stem cell niche providing a privileged site in the bone marrow that protects HSC from immune attack [90, 91].

Several mechanisms have been proposed through which Treg promote tolerance to stem cell grafts and exert their suppressive function. Treg can produce several cytokines of which interleukin-10 (IL-10), interleukin-35 (IL-35), and TGF-*β* have been demonstrated to be important for their suppressive function [92–95]. In the context of donor HSC engraftment, TGF-*β* seems to play a key role as it induces stem cell quiescence and promotes osteoblastogenesis that is required for the maintenance of the HSC niche [96]. Treg function is also mediated by cell-cell contact through different molecules such as LAG-3, CTLA-4, CD39, granzymes, and perforin, but it is still not clear which is the main mechanism that Treg use to control alloreactions against transplanted donor HSC in the engraftment phase [97–101]. In animal models where Treg adoptive transfer is used for GvHD prevention, Treg mainly exert their function in the very early phase after transplantation in MHC-independent manner suggesting that Treg interact with the host environment to promote tolerance [102].

While Treg adoptive transfer has been already studied in clinical trials for GvHD prevention, similar treatments need to be explored in the clinic in the setting of HCT engraftment and organ transplantation. In these contexts, concerns rise regarding the purity of the cells and the need of* in vitro* or* in vivo* activation. To partially avoid these issues, conditioning regimens for HCT have been studied aiming to favor donor chimerism and protect host Treg without the need of a cellular therapy. The combination of total lymphoid irradiation (TLI) with antithymocyte globulin (ATG) has been used as reduced intensity conditioning regimen (RIC) in HCT. TLI/ATG has been demonstrated to be effective in inducing stable mixed chimerism and favors immune tolerance [103]. After TLI/ATG treatment, the residual T cell pool is highly enriched in Treg and preclinical studies showed that in the TLI/ATG setting host-type Treg together with invariant natural killer T cells (iNKT) play a crucial role in protecting donor cells from host immune attack permitting the development of stable mixed chimerism and inducing tolerance to organ transplantation [104]. These concepts have been translated into the clinic in patients with hematological malignancies that were not eligible for conventional myeloablative conditioning. A first trial from Stanford University demonstrated feasibility of TLI/ATG approach in humans. In this trial, only 3% of 37 transplanted patients with matched related or unrelated donor developed GvHD ≥grade II [105]. In a subsequent follow-up study, authors demonstrated that patients conditioned with TLI/ATG that reached complete donor chimerism had a more favorable outcome due to better tumor control in the absence of GvHD [106]. These promising results have been confirmed by others suggesting that TLI/ATG conditioning can be a suitable approach for HCT of patients that are unfit for conventional myeloablative regimen due to its tolerogenic properties [107–109]. Because of these results, TLI/ATG approach is also studied in preclinical models and clinical trials are ongoing to induce tolerance toward solid organ transplantation [110–112].

Other authors are focusing their effort on Treg-based treatments that can directly enhance Treg function* in vivo*. The use of blocking antibodies against DNAX accessory molecule 1 (DNAM-1) activating receptor is one clear example as it has shown to improve Treg efficacy in controlling GvHD [113]. Similarly, anti-DNAM-1 treatment could favor tolerance towards allogeneic HSC engraftment. DNAM-1 is preferentially expressed on NK cells and CD8 T cells and has been involved in improved cytotoxic function of these cell types towards tumor cells expressing DNAM-1 ligands CD155 (poliovirus receptor) and CD112 (nectin-2). However, these ligands can also bind with higher affinity to the inhibitory receptors CD96 and the T cell immunoglobulin and ITIM domain TIGIT molecule. TIGIT is expressed on NK cells, activated and memory T cells, and Tregs and its function is correlated with IL-10 secretion and immune regulation [114–116]. After HCT, it has been found that TIGIT is upregulated on Treg and that infusion of DNAM-1-deficient Treg in allogeneic HCT models resulted in reduced GvHD compared to its wild type counterparts [117]. The lack of DNAM-1 expression on the donor T cell compartment was also shown to improve the expansion and suppressive function of donor-derived Treg. Thus, neutralization of DNAM-1 can favor tolerance to allogeneic HCT and reduce GvHD [117].

Another limitation to the use of Treg in stem cell engraftment is their paucity in the periphery of the donor. Several authors have studied different approaches for Treg* in vitro* or* in vivo* expansion in order to increase their number and availability maintaining their suppressive function. We recently reported that the administration of low doses of iNKT expands donor Treg* in vivo* in HCT models allowing for effective GvHD prevention [118]. Moreover, in a recent report, the agonistic antibody for TNFRSF25 resulted in Treg* in vivo* expansion [119]. Our group tested this antibody in HCT animal models demonstrating its striking efficacy in limiting GvHD lethality [120]. Even if these approaches are promising, they need to be studied in models suitable for investigating donor HSC engraftment to further promote their clinical application.

While a large amount of studies demonstrated Treg tolerogenic properties through different mechanisms, it is still not clear whether Treg play a relevant role in physiological HSC development and differentiation. The complex system through which lineage differentiation is regulated requires the presence of several regulatory cytokines including TGF-*β* [96]. Moreover, studies on FoxP3^−/−^ mice showed impaired lymphocyte differentiation suggesting that Treg may be involved in establishing the fate of HSC [121, 122]. Further studies on this topic could provide a better understanding of the complex interplays present in the bone marrow environment. These, together with more specific animal models of bone marrow engraftment and rejection, could provide the preclinical basis to further utilize Treg in the clinic for inducing tolerance to bone marrow and hematopoietic HSC grafts.

Interestingly, the production of TGF-*β* by Treg can suppress NK cell activation and, consequently, the presence of Treg is thought to limit NK cell mediated antitumor responses. Therefore, many studies have focused on reducing Treg numbers or their effects in order to accomplish higher antitumor responses. However, a recent study has shown that Treg can preferentially suppress host licensed NK cells after MCMV infection [123]. If that is the case, administration of Treg right after allogeneic HCT could also prevent HSC rejection mediated by host licensed NK cells leaving the host activated unlicensed NK cells to reach a stronger antitumor effect and higher allogeneic HSC engraftment.

## 5. Conclusion

During the last decades, many attempts have been made to improve donor engraftment after allogeneic HCT not only to accomplish stronger antitumor responses but also to be able to utilize this therapy when autologous HCT is not an option and when RIC is required. New conditioning strategies along with immune cell infusion therapies have greatly increased HCT options and currently HLA-matched or HLA-mismatched HCT from related and unrelated donors are common strategies making HCT feasible and available for most patients. Unfortunately, it is still difficult to find the suitable approach that allows for a good balance between donor engraftment and GvHD and that favors effective and rapid immune reconstitution. Therefore, new strategies are needed to bypass the limitations of allogeneic HCT. In this review, we have highlighted the important role of both NK and Treg in the regulation of HSC engraftment and outcome after HCT. Strategies that favor the potential benefit of NK cells, such as the selection of donor NK cell subsets, and that enhance the suppressive role of Treg could be used to exploit the advantages of each immune cell population towards HSC engraftment. The infusion of donor alloreactive NK cells and Treg could putatively lead to significantly higher allogeneic HSC engraftment and more importantly could protect individuals at earlier phases after transplantation due to accelerated immune reconstitution. These therapeutic approaches could also reduce GvHD incidence and lethality and increase cytotoxic responses leading to better protection from both cancer relapse and infections. Moreover, these strategies could allow for more flexible and safer HCT protocols such as reduced intensity conditioning in elderly patients and children with hemoglobinopathies and immune deficiencies. New discoveries about Treg and NK cells may help to build a new “designed” graft that allows for personalized HCT.

## Figures and Tables

**Table 1:** Treg adoptive transfer in clinical trials.

	HCT type	Clinical approach	Patients treated	Treg isolation	Treg number/kg	GvHD outcome
Trzonkowski et al., 2009 [78]	HLA-matched	T: aGvHD and cGvHD	2 (1 aGvHD and 1 cGvHD)	Magnetic separation FACS sorting *In vitro* expansion	cGvHD (1 × 10^5^) aGvHD (2 × 10^6^)	1 (cGvHD) response

Brunstein et al., 2011 [75]	Cord blood	P	23	Magnetic separation *In vitro* expansion	1 × 10^5^–3 × 10^6^	aGvHD II–IV (43%) aGvHD III-IV (17%) cGvHD (14%)

Di Ianni et al., 2011 [76]	Haploidentical	P	28	Magnetic separation	2 × 10^6^–4 × 10^6^	aGvHD (2/26) No reported cGvHD

Martelli et al., 2014 [77]	Haploidentical	P	43	Magnetic separation	2 × 10^6^	aGvHD (6/41) cGvHD (1/41)

Theil et al., 2015 [79]	HLA-matched (4) Cw mismatch (1)	T: cGvHD	5	Magnetic separation *In vitro* expansion	Median 2.36 × 10^6^	Response (2) Stable (3)

T: treatment; P: prevention.
